# Supplementary material for: Regional differences in short stature in England between 2006 and 2019: A cross-sectional analysis from the National Child Measurement Programme
Source: PLoS Med. 2021 Sep 28;18(9):e1003760. doi: 10.1371/journal.pmed.1003760 (PMC8478195; doi:10.1371/journal.pmed.1003760)
Supplement: S2 Text — Barts Charity grant proposal research analysis plan (2018). (DOCX) [file pmed.1003760.s002.docx]

**S2 Text. Barts Charity grant proposal research analysis plan (2018).**

**Research design**

**Aim:** Determine the national prevalence and regional clustering of stunting in childhood.

**Approach:** UK children have height and weight measured at age 4 years through the National Child Measurement Programme (NCMP), with very high national uptake (95%). NCMP data on weight, height, age and postcode from 2006-2018 will be obtained from the UK Data Archive and NHS Digital to map stunting (low height-for-age) against UK growth standards. We will evaluate the association between linear growth failure and poverty, using data on index of multiple deprivation by postcode, and determine whether findings vary over time. We will then compare local and national data to ascertain whether there is an excess of stunting in east London, before and after adjusting for deprivation.

**Sample size justification**

This aim will utilise national data collected between 2006-2018, which is freely available online at NHS Digital from 2013 onwards (<https://digital.nhs.uk/services/national-child-measurementprogramme/>) and at the UK Data Archive for data between 2006-2012 (goo.gl/SNBcNL). Data are compiled and quality checked by the Government Statistical Service, and linked to postcode, local authority, gender, ethnicity and deprivation data. We can therefore comprehensively plot stunting prevalence over time both geographically and against key demographic data. All children who had a valid height measurement and linked postcode data will be included in the analysis. Uptake nationally in the NCMP was 95% in 2016-17, with over one million children measured annually (at ages 4 and 10 years), meaning findings are highly representative of UK pre-school children.
