# Supplementary material for: Regional differences in short stature in England between 2006 and 2019: A cross-sectional analysis from the National Child Measurement Programme
Source: PLoS Med. 2021 Sep 28;18(9):e1003760. doi: 10.1371/journal.pmed.1003760 (PMC8478195; doi:10.1371/journal.pmed.1003760)
Supplement: S3 Text — Table A: Height (cm) by ethnicity regression results, boys (n = 2,946,560). Table B: Height (cm) by ethnicity regression results, girls (n = 2,819,147). Table C: Height (cm) by ethnicity and IMD regression results, boys (n = 2,946,560). Table D: Height (cm) by ethnicity and IMD regression results, girls (n = 2,819,147). IMD, index of multiple deprivation. (DOCX) [file pmed.1003760.s003.docx]

**S3 Text. SatScan model specification.**

A Bernoulli probability model for count data was used for these analyses. Bernoulli probability models are useful for binary data and where the outcome is relatively rare. We tested for high stunting clusters only, and limited identification of clusters to a maximum size of 10% of the eligible population, due to the large population size of the total sample. Monte Carlo randomisation was carried out by SatScan to obtain a p-value, with the number of simulations set at 999.

*Adjustment strategies*

Adjustment was carried out at the individual level, by obtaining weighted height estimates for each ethnicity and IMD category. We fit an ordinary least squares regression model using height data, running models separately for boys and girls, adjusted for age in months (Tables A1-A4). Coefficients were used to generate a height estimate weighted for ethnicity and one weighted for ethnicity and IMD for each child. These estimates were transformed into SDS and used to derive a stunting variable, which was then used in place of the unadjusted variable in SatScan spatial and temporal analyses.

Alternative adjustment strategy: This strategy involves separate analysis of multiple datasets with a log-likelihood ratio calculated for the pooled results (SatScan User Guide). Separate datasets for each ethnic group were analysed. Separate datasets for IMD quintile, rather than decile, were analysed to avoid including ten separate datasets in the model. We were unable to adjust for ethnicity and IMD simultaneously using this strategy, as SatScan limits the number of datasets to a maximum of twelve.

**Table A.** **Height (cm) by ethnicity regression results, boys (n=2,946,560).**

| **Measure** | **Coefficient** | **95% CI** |
| --- | --- | --- |
| Age in months | 0.55 | 0.54; 0.55 |
| Ethnicity (ref: white) |  |  |
| Mixed | 0.69 | 0.66; 0.71 |
| Black African, Black Caribbean and Black other | 2.61 | 2.59; 2.64 |
| Indian | 0.11 | 0.07; 0.14 |
| Pakistani & Bangladeshi | 0.38 | 0.36; 0.40 |
| Other ethnicity | -0.14 | -0.17; -0.12 |
|  |  |  |
| Constant | 77.19 | 77.11; 77.26 |

**Table B. Height (cm) by ethnicity regression results, girls (n=2,819,147).**

| **Measure** | **Coefficient** | **95% CI** |
| --- | --- | --- |
| Age in months | 0.55 | 0.55; 0.55 |
| Ethnicity (ref: white) |  |  |
| Mixed | 0.79 | 0.77; 0.81 |
| Black African, Black Caribbean and Black other | 2.98 | 2.96; 3.00 |
| Indian | -0.04 | -0.08; -0.01 |
| Pakistani & Bangladeshi | 0.16 | 0.14; 0.19 |
| Other ethnicity | -0.31 | -0.33; -0.28 |
|  |  |  |
| Constant | 75.98 | 75.90; 76.06 |

**Table C. Height (cm) by ethnicity and IMD regression results, boys (n=2,946,560).**

| **Measure** | **Coefficient** | **95% CI** |
| --- | --- | --- |
| Age in months | 0.54 | 0.54; 0.55 |
| Ethnicity (ref: white) |  |  |
| Mixed | 0.76 | 0.74; 0.79 |
| Black African, Black Caribbean and Black other | 2.83 | 2.81; 2.86 |
| Indian | 0.17 | 0.14; 0.20 |
| Pakistani & Bangladeshi | 0.63 | 0.61; 0.65 |
| Other ethnicity | -0.03 | -0.05; -0.00 |
| IMD (ref: 5) |  |  |
| 1 | -0.51 | -0.53; -0.49 |
| 2 | -0.28 | -0.31; -0.26 |
| 3 | -0.19 | -0.21; -0.16 |
| 4 | -0.11 | -0.13; -0.08 |
| 6 | 0.06 | 0.03; 0.08 |
| 7 | 0.11 | 0.09; 0.14 |
| 8 | 0.19 | 0.16; 0.21 |
| 9 | 0.26 | 0.24; 0.29 |
| 10 | 0.37 | 0.35; 0.40 |
|  |  |  |
| Constant | 77.24 | 77.16; 77.32 |

**Table D. Height (cm) by ethnicity and IMD regression results, girls (n=2,819,147).**

| **Measure** | **Coefficient** | **95% CI** |
| --- | --- | --- |
| Age in months | 0.55 | 0.55; 0.55 |
| Ethnicity (ref: white) |  |  |
| Mixed | 0.86 | 0.84; 0.88 |
| Black African, Black Caribbean and Black other | 3.18 | 3.16; 3.21 |
| Indian | 0.02 | -0.01; 0.05 |
| Pakistani & Bangladeshi | 0.39 | 0.37; 0.42 |
| Other ethnicity | -0.20 | -0.23; -0.18 |
| IMD (ref: 5) |  |  |
| 1 | -0.46 | -0.48; -0.43 |
| 2 | -0.24 | -0.26; -0.22 |
| 3 | -0.15 | -0.17; -0.12 |
| 4 | -0.09 | -0.11; -0.06 |
| 6 | 0.07 | 0.05; 0.10 |
| 7 | 0.13 | 0.11; 0.16 |
| 8 | 0.19 | 0.17; 0.22 |
| 9 | 0.27 | 0.24; 0.29 |
| 10 | 0.36 | 0.34; 0.39 |
|  |  |  |
| Constant | 76.01 | 75.93; 76.09 |
