# Supplementary material for: Regional differences in short stature in England between 2006 and 2019: A cross-sectional analysis from the National Child Measurement Programme
Source: PLoS Med. 2021 Sep 28;18(9):e1003760. doi: 10.1371/journal.pmed.1003760 (PMC8478195; doi:10.1371/journal.pmed.1003760)
Supplement: S1 Table — IMD, index of multiple deprivation. (DOCX) [file pmed.1003760.s004.docx]

**S1 Table. Ethnicity and IMD missingness by sample characteristics.**

| **Characteristic** | **Included**  **(sample with no missing ethnicity or IMD data) n=5,765,707** | **Excluded**  **(sample with missing ethnicity or IMD data) n=1,296,364** |
| --- | --- | --- |
| Short stature (<-2.00 SDS, %) | 1.92 | 1.96 |
| Very short stature (<-2.67 SDS, %) | 0.37 | 0.36 |
| Age in months |  |  |
| Mean (SD) | 59.90 (4.01) | 60.19 (3.99) |
| Height in cm |  |  |
| Mean (SD) | 109.60 (5.15) | 109.69 (5.13) |
| Sex (%) |  |  |
| Boys | 51.10 | 51.07 |
| Girls | 48.90 | 48.93 |
| Government Office region (%) |  |  |
| North East | 4.64 | 6.45 |
| North West | 11.89 | 22.34 |
| Yorkshire and the Humber | 10.71 | 7.83 |
| East Midlands | 8.68 | 7.50 |
| West Midlands | 11.51 | 9.66 |
| East of England | 12.16 | 7.26 |
| London | 17.25 | 8.92 |
| South East | 14.07 | 20.76 |
| South West | 9.10 | 9.28 |
| Index of Multiple Deprivation (%) |  |  |
| 1 | 13.82 | 14.45 |
| 2 | 12.44 | 11.08 |
| 3 | 11.10 | 9.94 |
| 4 | 10.04 | 9.21 |
| 5 | 9.32 | 9.14 |
| 6 | 8.87 | 8.78 |
| 7 | 8.42 | 8.79 |
| 8 | 8.58 | 8.89 |
| 9 | 8.71 | 9.32 |
| 10 | 8.71 | 10.40 |
| Time period (%) |  |  |
| 2006-10 | 21.58 | 37.02 |
| 2010-13 | 25.10 | 18.59 |
| 2013-16 | 26.09 | 24.08 |
| 2016-19 | 27.24 | 20.31 |
