# Supplementary material for: Regional differences in short stature in England between 2006 and 2019: A cross-sectional analysis from the National Child Measurement Programme
Source: PLoS Med. 2021 Sep 28;18(9):e1003760. doi: 10.1371/journal.pmed.1003760 (PMC8478195; doi:10.1371/journal.pmed.1003760)
Supplement: S2 Table — (DOCX) [file pmed.1003760.s005.docx]

**S2 Table. Population sex, Government Office Region and ethnicity in children, 2011-2012 (Census 2011 and ONS population projections).**

| **Characteristic** | **2011 Census % (n) /**  **Population projections 2012** |
| --- | --- |
| Sex % (n)^a^ |  |
| Male | 51 (934,518) |
| Female | 49 (893,092) |
| Government Office Region % (n)^b^ |  |
| North East | 5 (141,000) |
| North West | 13 (405,000) |
| Yorkshire and the Humber | 10 (307,000) |
| East Midlands | 8 (256,000) |
| West Midlands | 11 (337,000) |
| East of England | 11 (340,000) |
| London | 17 (509,000) |
| South East | 16 (507,000) |
| South West | 9 (283,000) |
| Ethnicity % (n)^c^ |  |
| White British and White Other | 77 (1,404,647) |
| Black African, Caribbean & Other | 5 (97,718) |
| Indian | 3 (50,500) |
| Pakistani & Bangladeshi | 6 (103,293) |
| Mixed | 6 (102,047) |
| Other | 4 (69,405) |

^a^Based on Census 2011 data for 5 to 7 year olds (http://www.nomisweb.co.uk/census/2011/DC2101EW/view/2092957699?rows=c_sex&cols=c_age)

^b^Based on data from population estimates for 5 to 9 year olds in 2012 (<https://www.ons.gov.uk/peoplepopulationandcommunity/populationandmigration/populationprojections/datasets/regionsinenglandtable1>)

^c^Based on Census 2011 data for 5 to 9 year olds (http://www.nomisweb.co.uk/census/2011/dc2101ew)
