# Supplementary material for: Regional differences in short stature in England between 2006 and 2019: A cross-sectional analysis from the National Child Measurement Programme
Source: PLoS Med. 2021 Sep 28;18(9):e1003760. doi: 10.1371/journal.pmed.1003760 (PMC8478195; doi:10.1371/journal.pmed.1003760)
Supplement: S3 Table — IMD, index of multiple deprivation; NCMP, National Child Measurement Programme; SDS, standard deviation score. (DOCX) [file pmed.1003760.s006.docx]

**S3 Table. Percentage of children with short stature (<-2.00 SDS) by NCMP school year and IMD decile (n=7,061,591).**

| NCMP year | IMD  Q1 | IMD  Q2 | IMD  Q3 | IMD  Q4 | IMD  Q5 |
| --- | --- | --- | --- | --- | --- |
| 2006-07 | 2.67  [2.55; 2.79] | 2.36  [2.23; 2.50] | 1.94  [1.82; 2.07] | 1.97  [1.85; 2.11] | 1.68  [1.56; 1.80] |
| 2007-08 | 2.64  [2.55; 2.73] | 2.24  [2.15; 2.34] | 2.05  [1.96; 2.16] | 1.78  [1.69; 1.88] | 1.53  [1.45; 1.61] |
| 2008-09 | 2.56  [2.47; 2.65] | 2.06  [1.97; 2.15] | 1.87  [1.79; 1.96] | 1.62  [1.54; 1.71] | 1.37  [1.30; 1.45] |
| 2009-10 | 2.54  [2.45; 2.62] | 2.14  [2.05; 2.23] | 1.90  [1.81; 1.99] | 1.59  [1.51; 1.68] | 1.49  [1.41; 1.56] |
| 2010-11 | 2.59  [2.50; 2.67] | 2.12  [2.04; 2.21] | 1.87  [1.79; 1.96] | 1.71  [1.63; 1.80] | 1.42  [1.35; 1.49] |
| 2011-12 | 2.46  [2.39; 2.54] | 2.01  [1.93; 2.09] | 1.83  [1.75; 1.91] | 1.68  [2.60; 1.76] | 1.49  [1.42; 1.57] |
| 2012-13 | 2.44  [2.36; 2.51] | 2.13  [2.05; 2.21] | 1.88  [1.80; 1.96] | 1.69  [1.61; 1.77] | 1.45  [1.38; 1.53] |
| 2013-14 | 2.40  [2.33; 2.48] | 2.02  [1.95; 2.10] | 1.80  [1.72; 1.88] | 1.73  [1.65; 1.82] | 1.46  [1.39; 1.54] |
| 2014-15 | 2.34  [2.26; 2.41] | 2.00  [1.92; 2.08] | 1.76  [1.68; 1.84] | 1.64  [1.56; 1.72] | 1.39  [1.32; 1.46] |
| 2015-16 | 2.32  [2.25; 2.39] | 2.01  [.94; 2.09] | 1.65  [1.58; 1.73] | 1.63  [1.55; 1.70] | 1.40  [1.33; 1.47] |
| 2016-17 | 2.23  [2.16; 2.30] | 1.97  [1.90; 2.05] | 1.73  [1.66; 1.81] | 1.57  [1.51; 1.65] | 1.41  [1.34; 1.48] |
| 2017-18 | 2.24  [2.17; 2.31] | 1.89  [1.82; 1.96] | 1.74  [1.67; 1.82] | 1.63  [1.56; 1.71] | 1.37  [1.30; 1.45] |
| 2018-19 | 2.21  [2.14; 2.29] | 1.89  [1.81; 1.97] | 1.74  [1.67; 1.82] | 1.53  [1.46; 1.61] | 1.46  [1.39; 1.54] |
