# Supplementary material for: Regional differences in short stature in England between 2006 and 2019: A cross-sectional analysis from the National Child Measurement Programme
Source: PLoS Med. 2021 Sep 28;18(9):e1003760. doi: 10.1371/journal.pmed.1003760 (PMC8478195; doi:10.1371/journal.pmed.1003760)
Supplement: S4 Table — IMD, index of multiple deprivation; SDS, standard deviation score. (DOCX) [file pmed.1003760.s007.docx]

**S4 Table. Logistic regression of short stature (<-2.00 SDS) by IMD and year, including IMD#year interaction (n=7,061,591^a^).**

| Measure | Odds Ratio | SE | 95% CI |
| --- | --- | --- | --- |
| IMD decile (ref: 1) |  |  |  |
| **2** | **0.85** | **0.02** | **0.82; 0.89** |
| **3** | **0.78** | **0.02** | **0.75; 0.82** |
| **4** | **0.76** | **0.02** | **0.72; 0.80** |
| **5** | **0.71** | **0.02** | **0.68; 0.75** |
| **6** | **0.65** | **0.02** | **0.61; 0.68** |
| **7** | **0.63** | **0.02** | **0.59; 0.66** |
| **8** | **0.58** | **0.02** | **0.55; 0.61** |
| **9** | **0.53** | **0.01** | **0.50; 0.56** |
| **10** | **0.50** | **0.01** | **0.47; 0.53** |
|  |  |  |  |
| **NCMP year** | **0.98** | **0.00** | **0.98; 0.99** |
|  |  |  |  |
| **IMD#year (ref: IMD D1)** |  |  |  |
| IMD D2#year | 1.00 | 0.00 | 1.00; 1.01 |
| IMD D3#year | 1.01 | 0.00 | 1.00; 1.01 |
| IMD D4#year | 1.00 | 0.00 | 1.00; 1.01 |
| IMD D5#year | 1.00 | 0.00 | 1.00; 1.01 |
| **IMD D6#year** | **1.01** | **0.00** | **1.00; 1.01** |
| **IMD D7#year** | **1.01** | **0.00** | **1.00; 1.01** |
| **IMD D8#year** | **1.01** | **0.00** | **1.00; 1.02** |
| **IMD D9#year** | **1.01** | **0.00** | **1.01; 1.02** |
| **IMD D10#year** | **1.01** | **0.00** | **1.00; 1.02** |
|  |  |  |  |
| Constant | 0.03 | 0.00 | 0.03; 0.03 |

^a^ Note: Sample includes all eligible children with IMD data.

Significant estimates are in bold.
