# Supplementary material for: Regional differences in short stature in England between 2006 and 2019: A cross-sectional analysis from the National Child Measurement Programme
Source: PLoS Med. 2021 Sep 28;18(9):e1003760. doi: 10.1371/journal.pmed.1003760 (PMC8478195; doi:10.1371/journal.pmed.1003760)
Supplement: S5 Table — IMD, index of multiple deprivation; SDS, standard deviation score. (DOCX) [file pmed.1003760.s008.docx]

**S5 Table. Short stature (<-2.00 SDS) clusters, unadjusted and adjusted for both ethnicity and IMD (n=5,765,707).**

| Cluster^a^: | Region | White ethnicity ^b^ % (n) | Cluster mean (SD) IMD | Population ^c^ | Short stature % (n) | RR ^d^ |
| --- | --- | --- | --- | --- | --- | --- |
| **Unadjusted** |  |  |  |  |  |  |
| **Leicester** | East Midlands | 41 (20,737) | 2.89 (1.97) | 50,088 | 2.8  (1,409) | 1.47 |
| **Great Yarmouth, Norwich** | East of England | 90 (25,098) | 3.68 (2.45) | 27,793 | 2.7  (759) | 1.43 |
| **Burnley, Rossendale, Hyndburn, Pendle, Blackburn with Darwen, Calderdale, Rochdale, Ribble Valley, Bury, Bradford, Bolton,** **Oldham,** **Chorley,** Craven, **Preston, Kirklees** | North West,  Yorkshire and the Humber | 68  (254,716) | 3.85 (2.79) | 377,305 | 2.5  (9,399) | 1.33 |
| Exeter | South West | 91  (11,159) | 5.39  (2.47) | 12,211 | 2.5  (305) | 1.30 |
| **North East Lincolnshire, North Lincolnshire, West Lindsey, Kingston upon Hull, East Lindsey, Lincoln** | Yorkshire and the Humber, East Midlands | 92 (95,781) | 3.86 (2.70) | 103,569 | 2.4  (2,31) | 1.28 |
| **Tower Hamlets** | London | 17  (5,505) | 2.05 (1.37) | 32,754 | 2.4  (789) | 1.26 |
| Plymouth | South West | 94  (23,105) | 4.38  (2.70) | 24,514 | 2.4  (585) | 1.25 |
| County Durham, Darlington, **Gateshead**, Sunderland, Hartlepool, Stockton-on-Tees, Richmondshire, **Newcastle upon Tyne**, South Tyneside, Middlesbrough | North East, Yorkshire and the Humber | 91 (179,181) | 3.99 (2.84) | 197,474 | 2.2  (4,411) | 1.17 |
| **South Staffordshire, Wolverhampton, Cannock Chase, Walsall, Stafford, Sandwell, Telford and Wrekin, Dudley, Lichfield, East Staffordshire, Birmingham, Tamworth, Wyre Forest, Stoke-on-Trent** | West Midlands | 62 (272,336) | 3.43 (2.66) | 439,577 | 2.2  (2,531) | 1.15 |
| King’s Lynn and West Norfolk, Breckland, South Holland, Fenland, Forest Heath, North Norfolk | East of England, East Midlands | 96  (63,514) | 5.20  (2.05) | 66,347 | 2.2  (1,439) | 1.13 |
| **Adjusted for ethnicity and IMD** | | | | | | |
| **Great Yarmouth** | East of England | 92  (10,641) | 3.69 (2.45) | 11,513 | 2.9  (336) | 1.43 |
| Blaby, Oadby and Wigston, **Leicester**, Hinckley and Bosworth, Charnwood | East Midlands | 62  (61,080) | 5.06 (3.05) | 98,592 | 2.6  (2,570) | 1.28 |
| **Newham, Tower Hamlets** | London | 17  (14,552) | 2.11  (1.11) | 85,168 | 2.6  (2,186) | 1.26 |
| Harrow*,* **Brent** | London | 27  (17,521) | 4.62  (2.26) | 65,822 | 2.5  (1,675) | 1.25 |
| **Burnley, Rossendale, Hyndburn, Pendle, Blackburn with Darwen, Calderdale, Rochdale, Ribble Valley, Bury, Bradford, Bolton, Oldham, Chorley**, Craven, **Preston, Kirklees** | North West,  Yorkshire and the Humber | 68 (254,716) | 3.85  (2.79) | 377,305 | 2.5 (9,309) | 1.22 |
| **North East Lincolnshire, North Lincolnshire** | Yorkshire and the Humber | 90  (35,805) | 4.31 (2.90) | 39,853 | 2.4  (971) | 1.19 |
| **Gateshead, Newcastle upon Tyne** | North East | 81  (32,907) | 3.85  (2.89) | 40,757 | 2.4  (966) | 1.16 |
| **South Staffordshire, Wolverhampton, Cannock Chase, Walsall, Stafford, Sandwell, Telford and Wrekin, Dudley, Lichfield, East Staffordshire, Birmingham, Tamworth, Wyre Forest, Stoke-on-Trent** | West Midlands | 62  (272,336) | 3.43 (2.66) | 439,577 | 2.2  (9,879) | 1.11 |
| Central Bedfordshire, Luton, North Hertfordshire, Milton Keynes, Bedford, Stevenage | East of England, South East | 70  (102,163) | 6.08 (2.78) | 146,434 | 2.2  (3,255) | 1.09 |

^a^Clusters are referred to in the text by the name of the first LA in the cluster description. These are determined by SatScan and represent the centre point of the cluster. Clusters are ordered from highest to lowest RR. LAs present in ethnicity adjusted model (Table 2) are presented in bold.

^b^Cluster white ethnicity % and mean IMD are derived from NCMP data for children in each cluster.

^c^Cluster population is the total population of NCMP children included in the analysis for each cluster.

^d^No 95% CI is calculated for RR as the method for identifying clusters is data-driven, and 95% CIs would be inappropriate.
