# Supplementary material for: Regional differences in short stature in England between 2006 and 2019: A cross-sectional analysis from the National Child Measurement Programme
Source: PLoS Med. 2021 Sep 28;18(9):e1003760. doi: 10.1371/journal.pmed.1003760 (PMC8478195; doi:10.1371/journal.pmed.1003760)
Supplement: S6 Table — (DOCX) [file pmed.1003760.s009.docx]

**S6 Table. Time period analysis of short stature (<-2.00 SDS) (n=7,062,071).**

| Cluster ^a^ | Region | White ethnicity **^b^**  % (n) | Cluster average IMD**^b^** | Population **^c^** | Short stature % (n) | RR **^d^** |
| --- | --- | --- | --- | --- | --- | --- |
| 2006 to 2010 |  |  |  |  |  |  |
| **Leicester** | East Midlands | 45  (5,605) | 2.90 (2.02) | 13,796 | 2.8  (387) | 1.39 |
| **Bradford, Calderdale, Pendle, Kirklees,** Leeds, **Burnley, Craven, Rossendale, Rochdale,** Harrogate**, Oldham**, **Hyndburn, Wakefield**, **Ribble Valley, Bury,** Barnsley, **Blackburn with Darwen, Tameside** | Yorkshire and the Humber, North West | 74  (77,236) | 4.06 (2.84) | 152,426 | 2.6  (3,995) | 1.33 |
| **East Staffordshire, Lichfield, Cannock Chase, South Derbyshire, Stafford, Derby, Tamworth, South Staffordshire, Walsall,** North West Leicestershire, Staffordshire Moorlands, **Stoke-on-Trent, Amber Valley,** Derbyshire Dales, North Warwickshire, Erewash, **Wolverhampton** | West Midlands, East Midlands | 82  (56,783) | 4.87 (2.98) | 79,771 | 2.6  (2,060) | 1.29 |
| East Lindsey**, North East Lincolnshire**, Boston, **West Lindsey**, **Lincoln, North Kesteven, North Lincolnshire,** South Holland, **South Kesteven, Kingston upon Hull, Newark and Sherwood, Bassetlaw, King’s Lynn and West Norfolk,** Peterborough, **Melton, Rushcliffe**, Rutland, **Doncaster,** **Mansfield, Gedling,** Fenland, East Riding of Yorkshire, **Bolsover, Nottingham, Ashfield, Rotherham**, North Norfolk | East Midlands, Yorkshire and the Humber, East of England | 91  (75,567) | 4.74 (2.89) | 124,754 | 2.4  (2,964) | 1.19 |
| Warrington, Halton, St. Helens, Trafford, Wigan, **Salford**, Knowsley, **Bolton,** Liverpool | North West | 86  (43,346) | 4.08 (2.99) | 80,444 | 2.3  (1,879) | 1.16 |
| 2010 to 2013 |  |  |  |  |  |  |
| **Great Yarmouth** | East of England | 93  (2,707) | 3.84 (2.44) | 2,937 | 3.7  (108) | 1.87 |
| Broxbourne | East of England | 84  2,216 | 6.36 (2.37) | 3,280 | 3.3  (108) | 1.67 |
| **Leicester** | East Midlands | 42  (4,937) | 2.92 (2.00) | 12,154 | 3.0  (361) | 1.51 |
| **North East Lincolnshire, North Lincolnshire, West Lindsey, Kingston upon Hull** | Yorkshire and the Humber, East Midlands | 88  (19,307) | 3.87 (2.81) | 22,889 | 2.8  (638) | 1.42 |
| **Burnley, Rossendale, Hyndburn, Pendle, Blackburn with Darwen, Calderdale, Rochdale, Ribble Valley, Bury, Bradford, Bolton, Oldham, Chorley, Craven, Preston, Kirklees, South Ribble, Manchester, Salford, Tameside** | North West, Yorkshire and the Humber | 67  (80,805) | 3.61 (2.72) | 143,006 | 2.5  (3,592) | 1.31 |
| **County Durham, Darlington**, **Gateshead**, **Sunderland, Hartlepool, Stockton-on-Tees, Richmondshire, Newcastle upon Tyne, South Tyneside, Middlesbrough** | North East, Yorkshire and the Humber | 91  (49,936) | 4.01 (2.84) | 64,712 | 2.4  (1,539) | 1.22 |
| **South Staffordshire, Wolverhampton, Cannock Chase, Walsall, Stafford, Sandwell, Telford and Wrekin, Dudley, Lichfield, East Staffordshire, Birmingham, Tamworth, Wyre Forest, Stoke-on-Trent** | West Midlands | 63  (62,365) | 3.47 (2.68) | 119,443 | 2.2  (2,633) | 1.13 |
| 2013 to 2016 |  |  |  |  |  |  |
| **Great Yarmouth** | East of England | 90  (2,889) | 3.69 (2.46) | 3,250 | 3.8  (124) | 2.02 |
| **Calderdale, Kirklees, Bradford, Rochdale, Burnley, Oldham, Rossendale, Pendle, Bury, Tameside, Hyndburn**, Leeds, **Blackburn with Darwen** | Yorkshire and the Humber, North West | 67  (72,336) | 3.79 (2.75) | 125,604 | 2.5  (3,122) | 1.35 |
| **County Durham, Darlington, Gateshead, Sunderland, Hartlepool, Stockton-on-Tees, Richmondshire, Newcastle upon Tyne, South Tyneside, Middlesbrough** | North East, Yorkshire and the Humber | 90  (53,019) | 3.94 (2.81) | 68,401 | 2.3  (1,583) | 1.24 |
| Torridge, North Devon, West Devon, Mid Devon, Teignbridge, **Exeter**, West Somerset, **Plymouth** | South West | 95  (21,447) | 5.04 (2.35) | 24,799 | 2.3  (559) | 1.20 |
| **Mansfield, Ashfield, Bolsover**, **Gedling, Newark and Sherwood**, North East Derbyshire, Chesterfield, Broxtowe, **Nottingham,** Amber Valley, **Bassetlaw, Rotherham**, Erewash**, Rushcliffe,** Derby, Derbyshire Dales, Sheffield, **Doncaster**, **Lincoln**, South Derbyshire, **Melton,** Barnsley, Charnwood, **North Kesteven**, North West Leicestershire, High Peak, **West Lindsey**, Staffordshire Moorlands, **East Staffordshire, Wakefield**, **South Kesteven,** **Leicester** | East Midlands, Yorkshire and the Humber, West Midlands | 81  (121,202) | 4.65  (2.91) | 172,153 | 2.2  (3,709) | 1.16 |
| Shropshire, **Telford and Wrekin, South Staffordshire, Wolverhampton, Wyre Forest, Dudley, Stafford, Newcastle-under-Lyme,** **Sandwell, Cannock Chase, Walsall**, Bromsgrove, Malvern Hills, **Stoke-on-Trent**, Worcester, **Birmingham** | West Midlands | 65  (77,098) | 3.60 (2.72) | 139,035 | 2.1  (2,939) | 1.13 |
| 2016 to 2019 |  |  |  |  |  |  |
| **Pendle, Burnley, Ribble Valley, Craven, Hyndburn, Bradford, Rossendale, Calderdale, Blackburn with Darwen, Rochdale, Bury, Preston, Chorley,** Lancaster, **Oldham, Kirklees, Bolton** | North West, Yorkshire and the Humber | 67  (68,081) | 3.91 (2.80) | 117,357 | 2.2  (2,625) | 1.25 |
| **Brent** | London | 26  (2,658) | 3.70 (1.68) | 11,399 | 3.2  (365) | 1.77 |
| **Blaby, Oadby and Wigston, Leicester, Hinckley and Bosworth** | East Midlands | 55  (12,040) | 4.63  (2.96) | 22,541 | 2.6  (597) | 1.46 |
| **South Staffordshire, Wolverhampton, Cannock Chase, Walsall, Stafford, Sandwell, Telford and Wrekin, Dudley, Lichfield, East Staffordshire, Birmingham, Tamworth, Wyre Forest, Stoke-on-Trent, Newcastle-under-Lyme** | West Midlands | 59  (64,847) | 3.50 (2.69) | 136,544 | 2.1  (2,868) | 1.17 |
| **Rotherham**, Sheffield, Chesterfield, **Bolsover, Doncaster** | Yorkshire and the Humber, East Midlands | 80  (40,736) | 3.94 (2.83) | 44,870 | 2.1  (960) | 1.18 |
| **Middlesbrough, Stockton-on-Tees**, Redcar and Cleveland, **Hartlepool,** **Darlington**, Hambleton, **Sunderland, County Durham,** Ryedale, Scarborough, **South Tyneside,** **Gateshead** | North East, Yorkshire and the Humber | 93  (57,178) | 4.11 (2.80) | 67,500 | 2.0  (1,377) | 1.12 |

^a^ Clusters are referred to in the text by the name of the first LA in the cluster description. These are determined by SatScan and represent the centre point of the cluster. Clusters are ordered from highest to lowest RR. LAs present in more than one time period are presented in bold.

^b^ Cluster white ethnicity % and mean IMD are derived from NCMP data for children in each cluster. Ethnicity and IMD were not available for all children used in these analyses, so estimates of White % and IMD average are based on available data.

^c^ Cluster population is the total population of NCMP children included in the analysis for each cluster.

^d^ No 95% CI is calculated for RR as the method for identifying clusters is data-driven, and 95% CIs would be inappropriate.
