# Supplementary material for: Regional differences in short stature in England between 2006 and 2019: A cross-sectional analysis from the National Child Measurement Programme
Source: PLoS Med. 2021 Sep 28;18(9):e1003760. doi: 10.1371/journal.pmed.1003760 (PMC8478195; doi:10.1371/journal.pmed.1003760)
Supplement: S7 Table — (DOCX) [file pmed.1003760.s010.docx]

**S7 Table. Very short stature (<-2.67 SDS) prevalence by sample characteristics (n=7,062,071).**

| Characteristic | Value | Stunting prevalence (%) |
| --- | --- | --- |
| Stunting (%) | N=25,669 | 0.36 [0.36; 0.37] |
| Sex (%) |  |  |
| Male | 51.10 | 0.33 [0.32; 0.34] |
| Female | 48.90 | 0.40 [0.39; 0.41] |
| Government Office Region (%) |  |  |
| North East | 4.97 | 0.41 [0.39; 0.43] |
| North West | 13.80 | 0.42 [0.41; 0.43] |
| Yorkshire and the Humber | 10.18 | 0.43 [0.42; 0.45] |
| East Midlands | 8.46 | 0.38 [0.37; 0.40] |
| West Midlands | 11.17 | 0.41 [0.39; 0.42] |
| East of England | 11.26 | 0.35 [0.33; 0.36] |
| London | 15.72 | 0.29 [0.28; 0.30] |
| South East | 15.30 | 0.31 [0.30; 0.32] |
| South West | 9.14 | 0.35 [0.33; 0.36] |
| Ethnicity (%) |  |  |
| White British and White Other | 62.02 | 0.36 [0.38; 0.39] |
| Black African, Black Caribbean & Other | 4.45 | 0.12 [0.11; 0.14] |
| Indian | 2.39 | 0.51 [0.50; 0.57] |
| Pakistani & Bangladeshi | 4.94 | 0.46 [0.45; 0.50] |
| Mixed | 4.32 | 0.29 [0.29; 0.33] |
| Other | 3.52 | 0.50 [0.50; 0.55] |
| Missing | 18.35 | 0.37 [0.36; 0.38] |
| Index of Multiple Deprivation (decile) (%) |  |  |
| 1 | 13.94 | 0.52 [0.51; 0.54] |
| 2 | 12.19 | 0.44 [0.43; 0.45] |
| 3 | 10.89 | 0.41 [0.39; 0.42] |
| 4 | 9.89 | 0.37 [0.38; 0.41] |
| 5 | 9.29 | 0.35 [0.34; 0.37] |
| 6 | 8.85 | 0.33 [0.31; 0.34] |
| 7 | 8.48 | 0.30 [0.29; 0.32] |
| 8 | 8.63 | 0.28 [0.27; 0.30] |
| 9 | 8.82 | 0.26 [0.25; 0.28] |
| 10 | 9.01 | 0.23 [0.22; 0.24] |
| Missing | 0.01 | 0.63 [0.13; 1.82] |
| Time Period (%) |  |  |
| 2006 – 2010 | 24.41 | 0.39 [0.38; 0.40] |
| 2010 – 2013 | 23.90 | 0.37 [0.36; 0.38] |
| 2013 – 2016 | 25.72 | 0.36 [0.35; 0.37] |
| 2016 – 2019 | 25.97 | 0.34 [0.33; 0.35] |
