# Supplementary material for: Regional differences in short stature in England between 2006 and 2019: A cross-sectional analysis from the National Child Measurement Programme
Source: PLoS Med. 2021 Sep 28;18(9):e1003760. doi: 10.1371/journal.pmed.1003760 (PMC8478195; doi:10.1371/journal.pmed.1003760)
Supplement: S8 Table — IMD, index of multiple deprivation; SDS, standard deviation score. (DOCX) [file pmed.1003760.s011.docx]

**S8 Table. Very short stature (<-2.67 SDS) clusters in England, 2006-07 to 2018-19, unadjusted, adjusted for ethnicity, and adjusted for ethnicity and IMD (n=5,765,707).**

| Cluster ^a^ | Region | White ethnicity ^b^  % (n) | Cluster average IMD ^b^ | Population ^c^ | Short stature % (n) | RR ^d^ |
| --- | --- | --- | --- | --- | --- | --- |
| Unadjusted |  |  |  |  |  |  |
| **Leicester** | East Midlands | 41  (20,737) | 2.89  (1.97) | 50,088 | 0.57 | 1.58 |
| **Pendle, Burnley, Ribble Valley, Craven, Hyndburn, Bradford, Rossendale, Calderdale, Blackburn with Darwen, Rochdale, Bury, Preston, Chorley**, Lancaster, **Oldham, Kirklees, Bolton, South Ribble** | North West,  Yorkshire and the Humber | 69  (277,899) | 3.95  (2.81) | 401,796 | 0.51 | 1.44 |
| **South Staffordshire,** **Wolverhampton,** **Cannock Chase,** **Walsall,** **Stafford,** **Sandwell, Telford and Wrekin, Dudley,** **Lichfield, East Staffordshire, Birmingham**, **Tamworth,** Wyre Forest, Stoke-on-Trent | West Midlands | 62  (272,336) | 3.43  (2.66) | 439,577 | 0.44 | 1.23 |
| **Scarborough, Ryedale, Redcar and Cleveland, Middlesbrough, East Riding of Yorkshire, Hambleton, York, Stockton-on-Tees, Hartlepool, Kingston upon Hull, Darlington, Harrogate, Selby, Sunderland, North Lincolnshire, Leeds, South Tyneside, County Durham, Richmondshire, North East Lincolnshire, Wakefield, North Tyneside, Doncaster, Gateshead** | Yorkshire and the Humber,  North East | 89.64 | 4.54  (2.99) | 553,409 | 0.42 | 1.17 |
| Adjusted for ethnicity |  |  |  |  |  |  |
| **Brent** | London | 25  (9,057) | 3.30  (1.55) | 35,997 | 0.63 | 1.59 |
| **Leicester** | East Midlands | 41  (20,737) | 2.89  (1.97) | 50,088 | 0.62 | 1.57 |
| **Burnley, Rossendale, Hyndburn, Pendle, Blackburn with Darwen, Calderdale, Rochdale, Ribble Valley, Bury, Bradford, Bolton, Oldham, Chorley, Craven, Preston, Kirklees, South Ribble,** Manchester, Salford | North West,  Yorkshire and the Humber | 67  (317,638) | 3.64  (2.74) | 476,894 | 0.53 | 1.36 |
| **Tower Hamlets, Newham** | London | 17  (14,552) | 2.11  (1.11) | 85,168 | 0.55 | 1.40 |
| **Dudley, Sandwell, Wolverhampton, Birmingham, Walsall, Wyre Forest** | West Midlands | 54  (177,266) | 2.96  (2.37) | 330,203 | 0.51 | 1.30 |
| **Scarborough, Ryedale, Redcar and Cleveland, Middlesbrough, East Riding of Yorkshire, Hambleton, York, Stockton-on-Tees, Hartlepool, Kingston upon Hull, Darlington, Harrogate, Selby, Sunderland, North** **Lincolnshire, Leeds, South Tyneside, County Durham, Richmondshire, North East Lincolnshire, Wakefield, North Tyneside, Doncaster, Gateshead** | Yorkshire and the Humber, North East | 90  (496,097) | 4.54  (2.99) | 553,409 | 0.43 | 1.11 |
| Adjusted for ethnicity and IMD |  |  |  |  |  |  |
| **Brent** | London | 25  (9,057) | 3.30  (1.55) | 35,997 | 0.6  (220) | 1.58 |
| **Leicester**, Oadby and Wigston | East Midlands | 43  (24,344)43 | 3.35  (2.43) | 56,081 | 0.6  (312) | 1.44 |
| **Pendle, Burnley, Ribble Valley, Craven, Hyndburn, Bradford, Rossendale, Calderdale, Blackburn with Darwen, Rochdale, Bury, Preston, Chorley**, Lancaster, **Oldham, Kirklees, Bolton, South Ribble** | North West,  Yorkshire and the Humber | 69  (277,899) | 3.95  (2.81) | 401,796 | 0.5  (2,001) | 1.31 |
| **Tower Hamlets, Newham, Hackney, Redbridge** | London | 23  (36,038) | 2.76  (1.76) | 156,295 | 0.5  (758) | 1.26 |
| **Dudley, Sandwell, Wolverhampton, Birmingham, Walsall, Wyre Forest** | West Midlands | 54  (177,266) | 2.96  (2.37) | 330,203 | 0.5  (1,523) | 1.20 |

^a^ Clusters are referred to in the text by the name of the first LA in the cluster description. These are determined by SatScan and represent the centre point of the cluster. Clusters are ordered from highest to lowest RR. LAs present in more than one model (unadjusted, adjusted for ethnicity and adjusted for IMD and ethnicity) are presented in bold.

^b^ Cluster white ethnicity % and mean IMD are derived from NCMP data for children in each cluster.

^c^ Cluster population is the total population of NCMP children included in the analysis for each cluster.

^d^ No 95% CI is calculated for RR as the method for identifying clusters is data-driven, and 95% CIs would be inappropriate.
