# Supplementary material for: Regional differences in short stature in England between 2006 and 2019: A cross-sectional analysis from the National Child Measurement Programme
Source: PLoS Med. 2021 Sep 28;18(9):e1003760. doi: 10.1371/journal.pmed.1003760 (PMC8478195; doi:10.1371/journal.pmed.1003760)
Supplement: S1 Fig — (DOCX) [file pmed.1003760.s013.docx]

**S1 Fig. Short stature clusters in England (London inset) 2006-07 to 2018-19, unadjusted.**


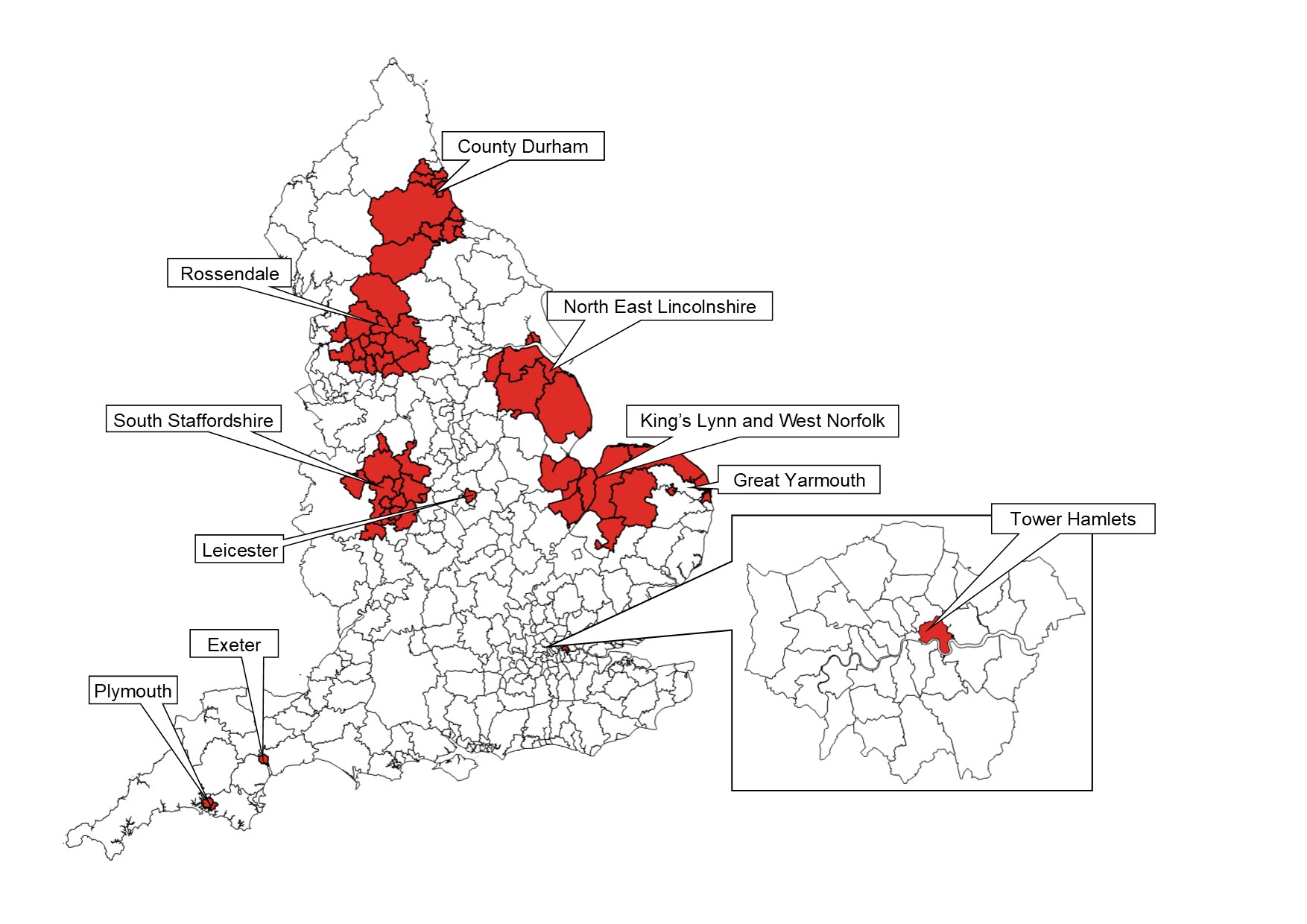


Note: Short stature clusters are in red. Map base layer is shapefile Local Authority Districts (December 2017) Full Clipped Boundaries in Great Britain, published by the Office for National Statistics and available at <https://geoportal.statistics.gov.uk/datasets/local-authority-districts-december-2017-full-clipped-boundaries-in-great-britain/explore?location=55.450000%2C-2.950000%2C5.64&showTable=true>.
