# Supplementary material for: Regional differences in short stature in England between 2006 and 2019: A cross-sectional analysis from the National Child Measurement Programme
Source: PLoS Med. 2021 Sep 28;18(9):e1003760. doi: 10.1371/journal.pmed.1003760 (PMC8478195; doi:10.1371/journal.pmed.1003760)
Supplement: Attachment — Submitted filename: 210701 Responses to reviewers.docx [file pmed.1003760.s015.docx]

| Comment | Response |
| --- | --- |
| Editor |  |
| 1)      Please revise your title according to PLOS Medicine's style. Please place the study design (e.g., "A retrospective cohort study”) in the subtitle (ie, after a colon). | We have changed the title to “Regional differences in short stature in England between 2006-2019: A cross-sectional analysis from the National Child Measurement Programme”. |
| 2)      In the abstract Methods and Findings: a)      Please structure your abstract using the PLOS Medicine headings (Background, Methods and Findings, Conclusions). b)      Please combine the Methods and results sections into one section, “Methods and findings”. c)      Please ensure that all numbers presented in the abstract are present and identical to numbers presented in the main manuscript text. d)      Please quantify the main results (with 95% CIs and p values). e)      Please include the important dependent variables that are adjusted for in the analyses. f)      In the last sentence of the Abstract Methods and Findings section, please describe the main limitation(s) of the study's methodology. g)      Please address the study implications without overreaching what can be concluded from the data; the phrase "In this study, we observed ..." may be useful. Mention only specific implications substantiated by the results. | a) We have restructured our abstract using the PLOS Medicine headings.  b) We have combined the sections Methods and Results.  c) We have confirmed all numbers presented in the abstract are correct.  d) We have added 95% CI to all estimates in the abstract.  e) We have stated that the main spatial analysis was adjusted for ethnicity, as this is the model presented in the main manuscript.  f) We have included as a final line in the results:  “This study was conducted cross-sectionally at an area level, and therefore we cannot make any inferences about the individual causes of short stature.”  g) We have amended our language, in particular in the conclusion, which now reads:  “In this study we observed a clear social gradient and striking regional variation in short stature across England, including a North-South divide. These findings provide impetus for further investigation into potential socioeconomic influences on height and the factors underlying regional variation.” |
| 3)      Author summary - At this stage, we ask that you include a short, non-technical Author Summary of your research to make findings accessible to a wide audience that includes both scientists and non-scientists. The Author Summary should immediately follow the Abstract in your revised manuscript. This text is subject to editorial change and should be distinct from the scientific abstract. Please see our author guidelines for more information: <https://eur01.safelinks.protection.outlook.com/?url=https%3A%2F%2Fjournals.plos.org%2Fplosmedicine%2Fs%2Frevising-your-manuscript%23loc-author-summary&amp;data=04%7C01%7C%7C63a8ab88583649fafaba08d92c3c28c8%7C569df091b01340e386eebd9cb9e25814%7C0%7C1%7C637589459168705865%7CUnknown%7CTWFpbGZsb3d8eyJWIjoiMC4wLjAwMDAiLCJQIjoiV2luMzIiLCJBTiI6Ik1haWwiLCJXVCI6Mn0%3D%7C1000&amp;sdata=SxnKzhwrhCMis7W1iNRUIHt5yaijO8TdcBm8fG8JzMI%3D&amp;reserved=0>. | We include an author summary in this revised version of the manuscript. |
| 4)      Did your study have a prospective protocol or analysis plan? Please state this early in the Methods section. a)      If a prospective analysis plan (from your funding proposal, IRB or other ethics committee submission, study protocol, or other planning document written before analyzing the data) was used in designing the study, please include the relevant prospectively written document with your revised manuscript as a Supporting Information file to be published alongside your study, and cite it in the Methods section. A legend for this file should be included at the end of your manuscript. b)      If no such document exists, please make sure that the Methods section transparently describes when analyses were planned, and when/why any data-driven changes to analyses took place. c)      In either case, changes in the analysis-- including those made in response to peer review comments-- should be identified as such in the Methods section of the paper, with rationale. | We have included two lines regarding the original analysis plan in the Methods section (p.5). We also include a section from the original grant proposal as supplemental material. We have not included the full document, as it contains information that is not appropriate to publish (such as names, addresses, phone numbers and financial information). For full transparency, we are attaching the original grant document for the editors and reviewers. |
| 5)      Your study is observational and therefore causality cannot be inferred. Please remove language that implies causality, such as impact. Refer to associations instead. For example, in the discussion section, replace the statement, “… both have a clear impact on height at the age of 4-5” with “… are associated with height at the age…” | We have edited the language to ensure causality is not implied. |
| 6)      Please add the following statement, or similar, to the Methods: "This study is reported as per the Strengthening the Reporting of Observational Studies in Epidemiology (STROBE) guideline (S1 Checklist)." The STROBE guideline can be found here: <https://eur01.safelinks.protection.outlook.com/?url=http%3A%2F%2Fwww.equator-network.org%2Freporting-guidelines%2Fstrobe%2F&amp;data=04%7C01%7C%7C63a8ab88583649fafaba08d92c3c28c8%7C569df091b01340e386eebd9cb9e25814%7C0%7C1%7C637589459168705865%7CUnknown%7CTWFpbGZsb3d8eyJWIjoiMC4wLjAwMDAiLCJQIjoiV2luMzIiLCJBTiI6Ik1haWwiLCJXVCI6Mn0%3D%7C1000&amp;sdata=rgWy7cjqhsS2hFuNFpkqFfe6OXRjWQ4Zdxe9oqX4mls%3D&amp;reserved=0> . When completing the checklist, please use section and paragraph numbers, rather than page numbers. | We have added the line: “This study is reported as per Strengthening the Reporting of Observational Studies in Epidemiology (STROBE) guidelines (Supplement A).” We have also included the STROBE checklist as supplemental material. |
| 7)      In the Methods and Results section: a)      Please provide 95% CIs and p values for all estimates. b)      When a p value is given, please specify the statistical test used to determine it. c)      Please present numerators and denominators for percentages, at least in the Tables | A) We have now included 95% CIs for all estimated percentages.  B) We have added a line in the methodology which discusses the way significance was determined for spatial analyses. We have removed p values as suggested by Reviewer 2, and included the level of statistical significance used in the text.  C) We now present numerators as well as percentages in the tables (with numerators, or total sample size, in the Table title). |
| 8)      For your Tables and figures, please define the abbreviations such as SD, IMD, NCMP, SE, CI | We have now defined these abbreviations. |
| 9)      Please use the "Vancouver" style for reference formatting and see our website for other reference guidelines. For example, six names should appear before et al. Please ensure that journal name abbreviations match those found in the National Center for Biotechnology Information (NCBI) databases, and are appropriately formatted and capitalized. <https://eur01.safelinks.protection.outlook.com/?url=https%3A%2F%2Fjournals.plos.org%2Fplosmedicine%2Fs%2Fsubmission-guidelines%23loc-references&amp;data=04%7C01%7C%7C63a8ab88583649fafaba08d92c3c28c8%7C569df091b01340e386eebd9cb9e25814%7C0%7C1%7C637589459168705865%7CUnknown%7CTWFpbGZsb3d8eyJWIjoiMC4wLjAwMDAiLCJQIjoiV2luMzIiLCJBTiI6Ik1haWwiLCJXVCI6Mn0%3D%7C1000&amp;sdata=e3G%2FEz39Dk2Nnskp%2BDNOWFTkAAGVb5d8eY7QOZhbyMg%3D&amp;reserved=0>. Please ensure that weblinks are current and accessible to date. a)      Six names should appear before et al, in the reference section. Please correct refs # 20, 29, 31 b)      Please include access dates for all weblinks and ensure that all weblinks are current and accessible. | We have reformatted all references in Vancouver style, using the Plos Medicine style in Endnote. We have also manually reviewed the reference list to ensure they were all properly formatted. |
| 10)     Please include line numbers in you next revision | We have now included line numbers. |
| Reviewer #1: |  |
| This is a very important study and the first to my knowledge that height of children from the NCMP program has been analysed separately from obesity alone and that has powerful socio-economic implications.  The study is well conducted appropriately statistically analysed and very well presented including the extra supplemental material, although the statistical process is one of spatial analysis, 'regional analysis' might be a more accessible term for to use for the title. | Thank you for your kind and useful comments. We have changed “spatial analysis” to “regional analysis” in the title. |
| I am uncomfortable with the use of the term 'stunting' here. Although the WHO does use this for height of children below -2 standard deviations it also includes in the definition of not achieving their full adult high potential. That is very difficult to infer from this study given the information presented here, hence in my view the term 'short stature' is a more preferable term to stunting as there is no evidence of a long term growth decrement and a reduction of adult height compared with genetic or familial expectations. The simple explanation could be different growth patterns. We know already that Afro-Caribbean childen are more physiologically advanced than Caucasians. | Thank you for this perspective. We discussed at length the implications for the use of “stunting” versus “short stature” as a group, including the ability to compare with international data. We ultimately agree with your view and have made the decision to refer to “short stature” throughout the paper. We still reference stunting in the introduction and discussion, to contextualise our findings within the international literature. |
| I have no additional comments to make about the ethical or analytical processes. These appear to be well done and I am not an expert in spatial analysis. | Thank you. |
| Data of the proportions of the sample each of the region are presented in table 1 together with the ethnicity breakdown. It would be helpful for comparative purposes if the figures from the 2011 population survey were included to validate these values. | We have included data from the 2011 census for sex and ethnicity in children aged 5 to 7 and 5 to 9 years respectively. We have included these as supplemental material (Supplemental Table E) so as not to crowd Table 1. We were unable to find the same information for government office region, so we include projections for the year 2012 calculated in 2012. All of these show high agreement between the population and our sample characteristics. Although there appears to be a discrepancy in the percentage of white children, this disappears once calculating percentages of available data (not including missing): 76% White, 5% Black, 3% Indian, 6% Pakistani and Bangladeshi, 5% mixed and 4% other ethnicities. |
| The changes in the IMD quintile over time as presented in figure 2 is very important information and perhaps reasons that this has occurred could be explored in more detail in the discussion section other than putting this down to obesity. Presumably with the NCMP data you can affirm or deny that conclusion. | Thank you for this suggestion. There appear to be two main possible explanations. In the original manuscript we included what we consider to be the most likely, given that the prevalence of obesity in this age group has also changed rapidly over the same time period and there is a clear social gradient, where overweight is not declining among the most deprived and may in fact be increasing. We have now included an additional hypothesis relating to evidence from the 2020 follow-up to the Marmot Review, that in the decade to 2020, children from low-income families have demonstrated better development and educational outcomes at age 5-6 in low-income areas than those in high-income areas. We believe analysis of NCMP data may lend itself to further exploration of this question, however we understand the relationship between BMI and height is complex. As such, this deserves a detailed analysis which is beyond the scope of this paper. |
| Height determinations are made according to the UK 1990 British references. Reference 11 is correct but WHO data are used until age 4 then UK 1990 thereafter. These are built exclusively on the white British population **therefore further adjustment needs to be made to represent all the ethnic minorities present**. It is little surprising that those of Asian background are significantly shorter and those of Afro-Caribbean taller. That discussion needs to be expanded. | Thank you, we agree that this is an important limitation. We have added the following to our limitation section:  “The population reference used (UK-WHO) is constructed using data from the UK 1990 for the age group analysed, which was in turn developed using data from white British children. This may limit its accuracy in children of other ethnicities.”  We also agree that the question of ethnicity is an important one and one which deserves further discussion. However we feel that this paper cannot fully address this issue. We have, however, expanded the discussion to include some of these complexities (p.16) |
| The presentation of the proportion below the second centile by ethnic background is important. However, there are no references to known heights of adults from relative ethnic backgrounds presented and perhaps a further sub-analysis based on this information might be helpful for further interpreting the findings. Reference to the country of origin might need to be made. | Thank you for this comment.  As far as we are aware, the only contemporary national data of adult height disaggregated by self-reported ethnicity in the UK come from the Active Lives Adult Survey (https://www.ethnicity-facts-figures.service.gov.uk/health/diet-and-exercise/overweight-adults/3.0#download-the-data).  The BMI data by ethnicity are reported but height data are not in the public domain. We have not explored an access request to these data because the relationship is complex between the heights of 4-5 year old children in the NCMP and the sample of adults in the ALAS. There are no data on country of birth/origin in either dataset, and it is possible that many adults in the ALAS have migrated to the UK from countries with different constraints on growth. Therefore, differences in average height of adults of different social/ethnic backgrounds currently living in the UK do not necessarily reflect the final growth potential of children born in the UK in the 21^st^ century.  This certainly warrants much more detailed exploration, which we feel is not possible within the scope of this paper, but we are currently exploring this in other work. |
| Furthermore, the data used to produce the 1990 charts was collected from a number of sources and at that stage there already was a north south divide evident in heights of children within the UK. The 1995 Freeman and Cole paper in Archives of Disease in Childhood 1995; 73: 17-24 discusses differences in heights across the country with Newcastle infants being lighter and Leeds school children being shorter than those in the south. The male-female disparity was different however. | Thank you. We have included this information and reference. |
| On page 17 there is a comment that those without an identifiable cause of stunting would be labelled as idiopathic short stature. However, a simple height measurement below the second centile cannot really define either idiopathic short stature or stunting. The value of height screening has been much debated but there is significant evidence in particular for Dutch and Finnish studies as discussed in the paper that a stepwise approach can be taken to identify short children with significant pathology but often requires repeat measurements and knowledge of the parents' heights. Hence, if an argument was constructed to use height measurement to detect socioeconomic deprivation and to detect pathology more often than the NCMP programme could allow, then the discussion needs to reflect that. I don't think that the final line of the abstract conclusion, 'Many children in the most deprived areas of the country may be failing to reach their full growth potential' can be fully justified based on the information presented here without additional expansion. Overall, though this is an important paper and needs to be presented in the public domain. | We have edited the relevant sections about idiopathic short stature to reflect your comments as well as the comments from Reviewer 2. We have included the more strict cut off of <-2.67 which is generally used in England to refer a child for investigation.  We have also added to the discussion around growth screening in the UK (p.18). Unfortunately, screening for growth disorders is not considered to be a priority in the UK. The NCMP is inadequate for picking up growth disorders, in particular as its focus is obesity and population monitoring (rather than individual screening).  We also agree that it is not possible with these data to justify the conclusion that children may be failing to reach their growth potential and have edited the final line of the abstract. |
| Reviewer #2: |  |
| The authors describe a spatial analysis of stunting in English children aged 5, based on the NCMP. The paper is excellent, with an extremely large dataset imaginatively analysed and carefully interpreted. I have some mainly minor comments on the study analysis, presentation and interpretation.  1.      I should declare my conflict of interest, having constructed (with Mike Preece and Jenny Freeman) the British 1990 height reference, which at age 5 equates to the UK-WHO reference. The stunting rate should be close to 2.28%, the proportion below -2 SDS, so the paper provides a large-scale validation of our chart construction skills.  2.      I am pleased to see that the mean stunting rate of 1.93% is close to the expected value, though the apparent sex difference in rates probably reflects a bias in the reference - see later. | Thank you for taking the time to review our manuscript and for your detailed and helpful comments. We hope we have addressed each one fully and adequately. |
| 3.      Abstract. Note that the UK-WHO is a growth reference, not a growth standard. | Thank you for this correction. We have amended the abstract. |
| 4.      When describing the national state school coverage, it would be useful to say what percentage of children aged 5 are in state schools. | We have now included the percentage of children attending state-maintained primary schools in 2010. |
| 5.      What is a "suppressed" version of the NCMP dataset? | We apologise for not clarifying this in the text. The ‘suppressed’ datasets are the datasets NHS digital and Public Health England make publicly available – these contain a great amount of data, however, for the sake of anonymisation, cases are ‘suppressed’ (removed from the dataset) when there are fewer than 6 cases in any published category (such as categories based on BMI – underweight, severely obese etc). In practice, this impacts smaller local authorities, and tends to impact children who are at the extremes (of BMI for example).  We have changed the wording of this sentence to clarify (p.5):  “NHS Digital makes a limited version of the NCMP dataset publicly available for analysis, which does not contain data that could lead to the identification of any child.” |
| 6.      Why was the cut-off of ±5 SDS used for data cleaning, as opposed to say ±4? | We were keen to keep as many observations as possible, and due to the large sample size, and internal validations conducted by the NCMP, we felt it was plausible for some children to be <-4 or >4 SDS without this being down to measurement error. WHO generally recommend a cut-off for anthropometric data of -5 or -6 SD as implausible (https://www.who.int/publications/i/item/9789241515559) |
| 7.      The definition of severe stunting used in the paper is wrong (page 5). The cut-off is -2.67 not -2.65 SDS, following the convention proposed by me (Cole TJ. Do growth chart centiles need a face lift? BMJ 1994;308:641-642). Thus the nominal severe stunting rate is 0.38% rather than 0.40%. The whole sentence needs redrafting, as it is not true to say it is "often" used as a referral cut-off, it is _the_ cut-off for defining short stature in the UK. See for example <https://eur01.safelinks.protection.outlook.com/?url=https%3A%2F%2Fwww.rcpch.ac.uk%2Fsites%2Fdefault%2Ffiles%2FBoys_2-18_years_growth_chart.pdf&amp;data=04%7C01%7C%7C63a8ab88583649fafaba08d92c3c28c8%7C569df091b01340e386eebd9cb9e25814%7C0%7C1%7C637589459168705865%7CUnknown%7CTWFpbGZsb3d8eyJWIjoiMC4wLjAwMDAiLCJQIjoiV2luMzIiLCJBTiI6Ik1haWwiLCJXVCI6Mn0%3D%7C1000&amp;sdata=DtOwq853j33QSQIAzv0%2Bwdtymr6nJ2%2F%2FoDgUIR7cVpU%3D&amp;reserved=0>. This relates to comments later about idiopathic short stature - they should relate to the -2.67 not the -2 cut-off. | Thank you for bringing this to our attention. We have re-analysed the data and present these new correct results in the revised manuscript. |
| 8.      The Statistical Analyses section does not include a sentence specifying the level of significance being used. Despite this p-values appear throughout the text, mainly p < 0.001. I suggest omitting most if not all of the p-values, as the sample size is enormous and the standard errors tiny, ensuring significance even for very small differences. The confidence intervals provide the same information more effectively. | We have removed p values and log-likelihood ratios from the tables and added further explanation on these in the methods and results. Namely, a description of the methodology for statistical significance in the methods section (p.7):  “We present all statistically significant clusters (threshold p<0.05), determined by log-likelihoods greater than a critical value established for each model through Standard Monte Carlo and Gumbel approximation.”  And this line in the results section, discussing spatial analyses:  “All clusters identified in this model were highly significant (p<0.001).” |
| 9.      In Figure 2 it would be useful to reverse the IMD legend so it's in the same direction as the graphic. What does the title "Equiplot (13)" mean? | Thank you for this suggestion. We have reversed the order of the IMD legend.  The (13) in the Equiplot title was the citation for the developers of the plot style and programme. However, as this is cited elsewhere in the paper, we have removed it to avoid confusion. |
| 10.     The cluster results in Figure 3 are very interesting, particularly the position of Leicester, which tops the list but appears as a tiny blob in the figure. I suggest labelling the clusters, primarily for non-British readers. **It would also be worth discussing in a bit more detail exactly why Leicester should achieve this dubious distinction**. Clearly it has a high proportion of Asian children, but ethnicity is adjusted for so it ought not to be the explanation. Has Leicester featured prominently in other indicators of deprivation? | Thank you for this comment. We have now included a reference to a paper which describes a ‘Midlands effect’ in short stature and other child health outcomes (though using data of a sample of exclusively Caucasian children participating the Millennium Cohort Study). Leicester itself is 32^nd^ from the bottom out of a total 317 local authorities in terms of average IMD, but the socioeconomic reasons for its place at the very top of the list of clusters in the NCMP analysis are not completely clear (just as the ‘Midlands effect’ is not fully understood). It seems likely that there is a combination of interacting residual socioeconomic/ethnic factors – but what these are we cannot answer in detail from these data.  As suggested we have labelled the clusters. |
| 11.     The title for Table 2 looks odd, saying it's adjusted both for ethnicity and for ethnicity and IMD. I suspect just the former is true. The table gives the stunting rate and RR to two decimal places, which is appropriate, but giving the white ethnicity percentage to two decimal places is not. It aims to distinguish between clusters like Brent, where 25% of children are white, and Great Yarmouth, where 90% are white. These percentages can given as whole numbers, and greater precision is unnecessary. This should apply throughout the text as well as the table. See my guidelines on the presentation of numerical information at <https://eur01.safelinks.protection.outlook.com/?url=http%3A%2F%2Fadc.bmj.com%2Fcgi%2Fcontent%2Ffull%2Farchdischild-2014-307149&amp;data=04%7C01%7C%7C63a8ab88583649fafaba08d92c3c28c8%7C569df091b01340e386eebd9cb9e25814%7C0%7C1%7C637589459168705865%7CUnknown%7CTWFpbGZsb3d8eyJWIjoiMC4wLjAwMDAiLCJQIjoiV2luMzIiLCJBTiI6Ik1haWwiLCJXVCI6Mn0%3D%7C1000&amp;sdata=K9g0IxdN77xLlI4SiNIjtFKQtN4kRGWaSH81y3fZMUs%3D&amp;reserved=0>. Also in Table 2 I suggest omitting the log-likelihood ratio and p-value columns, as they are respectively uninterpretable and uninformative. Note too that log-likelihood ratios to two decimal places are grossly over-precise. Table 1 would also be better with fewer decimal places. | Thank you for these guidelines.  We have changed the title for table 2 to make it clearer and reviewed all other table titles.  We have edited all percentages throughout text and tables to present them as whole numbers. Some exceptions are made when two decimal points make the percentage more informative, for example, percentages of children with short stature or very short stature.  We have also removed log-likelihood and p values from the spatial analyses tables. |
| 12.     Coming back to the sex difference, the stunting rates by sex are 2.09% for girls and 1.77% for boys. The nominal rate is 2.28%, so it's not so much that girls are high as that boys are low. But in fact the differences are small - the corresponding z-scores are -2.10 and -2.04, which equate to 2-5 mm deviation from the expected cut-offs. The Discussion states that this sex difference has not previously been reported in a high income setting, but it is almost certainly not generalisable. It is much more likely a bias in the reference as originally constructed. It may be worth mentioning the BMJ letter that we published soon after publishing the reference: Preece MA, Freeman JV, Cole TJ. Sex differences in weight in infancy - Published centile charts for weight have been updated. BMJ 1996;313:1486. It referred to infancy not age 5, but it indicates the care needed to avoid building a sex bias into the reference. | Thank you for highlighting this very important point. We have edited the text to reflect these comments. We have included a discussion of sex differences but have removed the suggestion that these data in particular reflect an important public health finding. |
| 13.     The second paragraph of the Discussion refers to idiopathic short stature, but as mentioned above it needs to be made clear that this diagnosis formally requires evidence of severe stunting, i.e. height SDS < -2.67. The same idea is picked up at the foot of page 17, where it is suggested that stunting is not recognised as an indicator of deprivation. | We have edited our language to reflect that we are referring to children generally in the community who may not have been investigated for short stature. The paragraph has been changed to: “Many of these children may in fact be particularly disadvantaged, unhealthy and failing to thrive.”  We have also added to the discussion around investigation of short stature in the UK, and, as you point out, that this investigation is currently triggered by a height SDS of <-2.67. |
| 14.     The scale of the dataset leads to some very powerful trends. The first paragraph of the Discussion states—correctly—that "Deprivation was linearly related to stunting", yet this statement holds even when applied to severe stunting, where in supplemental table C the severe stunting rate by IMD falls, perfectly monotonically, from 0.55 to 0.24 across the ten categories. I find this astonishing, and it calls to mind the famous phrase of James Tanner: "Growth as a mirror of the condition of society" (published as Tanner JM. Growth as a mirror of the condition of society: secular trends and class distinctions. Acta Paediatr Jpn 1987;29:96-103). This paper would be worth citing. | Thank you. As suggested, we have included a statement about the social gradient of severe stunting in the discussion, and we have included a reference to Tanner’s words (Discussion, p.15). |
| 15.     The reference to the dual burden and the suggestion that increasing obesity is fuelling a reduction in stunting in the more deprived is well made. | Thank you. |
| 16.     The paper lists its limitations, but I disagree that the last two mentioned are valid limitations. "As such, we will miss children with faltering growth whose height is crossing centiles and children whose height is above -2 SDS but who are below their target height as predicted by parental heights." Cross-sectional data cannot be expected to provide evidence of centile crossing, and the chosen definition of stunting does not involve parental height. I recommend removing the sentence. | Thank you. We agree and have removed this sentence. |
| 17.     A few minor comments on the Supplemental materials. The regression results should specify the units as cm. I suggest omitting the SE columns as they are so tiny (and the SE for the constant in Table A3 needs some attention). I found the table titles odd, a mix of letters and numbers with no obvious structure.  Tim Cole | Thank you, we have made these edits. |
| Reviewer #3: |  |
| This is a well-conducted spatial analysis of child stunting in England between 2006-2019. The study design, dataset, statistical methods and analyses, and presentation (tables and figures) and interpretation of the results are mostly adequate and of a good standard. The stats analyses in the supplementary information are very thorough and comprehensive with missing data issue particularly well addressed. Only a couple of minor points needing attention. | Thank you for taking the time to review our manuscript, and for your helpful suggestions. |
| 1) Table 2. Not clear what exactly was adjusted for. In the title, it says 'adjusted for ethnicity and adjusted for ethnicity and IMD' but in the table, there is only a line saying 'Adjusted for ethnicity'. Can authors please clarify this? | Thank you for bringing this to our attention. We have now clarified that Table 1 adjusts only for ethnicity. |
| 2) Time trends of stunting hot spots are striking as shown in Supplementary Figure F. Maybe authors can move Figure F into the main paper as it's of public health interest and give it a bit more discussion on the changes over time and the reasons behind these changes. | We have added Supplementary Figure F to the main paper, and we have added further to the discussion on these changes in p.17. |
