# Supplementary material for: Regional differences in short stature in England between 2006 and 2019: A cross-sectional analysis from the National Child Measurement Programme
Source: PLoS Med. 2021 Sep 28;18(9):e1003760. doi: 10.1371/journal.pmed.1003760 (PMC8478195; doi:10.1371/journal.pmed.1003760)
Supplement: Attachment — Submitted filename: 210728 Responses to reviewers RR2.docx [file pmed.1003760.s016.docx]

| Comment | Response |
| --- | --- |
| Editor |  |
| 1) Thank you for providing your STROBE checklist. Please replace the page numbers with paragraph numbers per section (e.g. "Methods, paragraph 1"), since the page numbers of the final published paper may be different from the page numbers in the current manuscript. | We have amended the STROBE checklist to now include section and paragraph. |
| Reviewer 1 |  |
| This is now a fantastic paper. Congratulations to the authors and for all the revisions. I recommend publication. | Thank you for your kind comments and suggestions. |
| Only 2 minor changes suggested:  Abstract P2 line 11 : should be ....heights from a total of 7xxxxxx children were analysed. | Thank you for this suggestion. We have amended the abstract. |
| Discussion p16 line 3:A 2015 analysis ...... children in the midlands were most likely to be to be shorter than SDS-1. I'm not completely clear what this means  Good luck! | We have re-worded this section to make it clearer. It now reads: “…where children in the Midlands were more likely to be shorter than -1 SDS, compared to children in other regions.” |
| Reviewer 2 |  |
| The authors have responded very positively to my suggestions. I have a few more minor comments for them to consider. | Thank you for reviewing our manuscript again and for your helpful comments and suggestions. |
| 1. The Discussion refers to the stunting rate in an international context, but it should be remembered that the British 1990 reference is not widely used internationally. This needs to be emphasised, and it would also be worth giving the rate as based on the WHO standard/reference. For girls the -2 SDS cut-off at 60 months is exactly the same (99.91 cm) for UK90 and WHO, but for boys it is 100.7 cm for WHO as against 100.5 for UK90. With a higher boys WHO cut-off there ought to be more WHO stunting, and I estimate the boys rate with WHO as 1.93% as against 1.77% with UK90. You can probably calculate it directly. | Thank you. This is a very good point. We are unfortunately currently unable to directly calculate the WHO standards rate using the final sample, as our colleague at Public Health England who has access to the dataset is on leave until September. We previously calculated these rates using an uncleaned version of the sample, where rate for boys was 1.74% using the UK90 and 1.93% using the WHO. I state these here to show that you are most likely correct in your estimation. We have expanded on this in paragraph 6 of the discussion (see next comment). |
| 2. This comparison between UK90 and WHO is also useful for interpreting the sex difference, in that the UK90 and WHO cut-offs are broadly the same so that perhaps the girls' excess is more genuine that I previously thought. Note that reference 25 (mentioned on page 16) refers explicitly to weight in infancy, and hence it is not directly relevant to height at age 5 - I referred to it just to highlight how tricky it can be to estimate growth centiles. | We have reworded paragraph 6 in the discussion to emphasise some of these points (including your previous point regarding the differences between the WHO and UK90 references):  “We found a statistically significantly higher prevalence of short stature in girls compared to boys. Population data on sex differences in short stature are useful, as existing data come from analyses of referral patterns to growth disorder clinics and are therefore subject to selection bias. Evidence from the USA suggests that boys are referred more readily and investigated more thoroughly than girls [25, 26]. Short stature is more common in boys than girls in low- and middle-income countries and it has been postulated that this is due to higher rates of adverse birth outcomes and increased vulnerability to infection and other morbidity during infancy in boys [27, 28]. These data might suggest that this is not a reasonable explanation for sex differences in referral patterns in the UK setting, as the incidence of adverse birth outcomes and childhood infections are higher for boys in the UK [29, 30]. It should be noted that the use of UK90 growth references means that rates of short stature in girls and boys will be slightly different to those calculated using the WHO standards, which are used in most of the international literature. This may explain some of the discrepancy observed between our results and international findings.” |
| 3. I note that the acronym SDS is used with the -2.67 cut-off but not with -2.0. I suggest harmonising its usage. | Thank you for flagging this. These have been harmonized. |
| 4. In Table 2 just one decimal place would be better for the cluster means. | We have changed the cluster means to one decimal place. |
| 5. The phrase 'short-statured children' appears on page 3, which might be better as 'children with short stature'. The later bullet point starting 'Short stature' should either omit 'also' or else reverse the sentence. | We have changed these to “children with short stature.” |
| 6. The maps of the clusters look very good, but I wondered if they could use colour more effectively, e.g. by adding a colour scale to reflect the % of white children in each cluster - just a thought, but it would emphasise how heterogeneous the ethnicity mix across the clusters.  Tim Cole | Thank you for this suggestion. We tried these maps out, and while they are visually interesting, we worried the main message would get dampened with the extra information about ethnicity. |
| Reviewer 3 |  |
| Many thanks authors for their effort to improve the manuscript. I am satisfied with the response and revision. No further issues needing attention. | Thank you for your review and helpful comments. |
